# Efficient production of α-acetolactate by whole cell catalytic transformation of fermentation-derived pyruvate

**DOI:** 10.1186/s12934-019-1271-1

**Published:** 2019-12-29

**Authors:** Robin Dorau, Lin Chen, Jianming Liu, Peter Ruhdal Jensen, Christian Solem

**Affiliations:** National Food Institute, Technical University of Denmark, 2800 Kgs. Lyngby, Denmark

**Keywords:** Biocatalysis, *Lactococcus lactis*, Metabolic engineering, Process engineering, Diacetyl, Lactose

## Abstract

**Background:**

Diacetyl provides the buttery aroma in products such as butter and margarine. It can be made via a harsh set of chemical reactions from sugarcane bagasse, however, in dairy products it is normally formed spontaneously from α-acetolactate, a compound generated by selected lactic acid bacteria in the starter culture used. Due to its bacteriostatic properties, it is difficult to achieve high levels of diacetyl by fermentation. Here we present a novel strategy for producing diacetyl based on whole-cell catalysis, which bypasses the toxic effects of diacetyl.

**Results:**

By expressing a robust α-acetolactate synthase (ALS) in a metabolically optimized *Lactococcus lactis* strain we obtained a whole-cell biocatalyst that efficiently converted pyruvate into α-acetolactate. After process optimization, we achieved a titer for α-acetolactate of 172 ± 2 mM. Subsequently we used a two-stage production setup, where pyruvate was produced by an engineered *L. lactis* strain and subsequently used as the substrate for the biocatalyst. Using this approach, 122 ± 5 mM and 113 ± 3 mM α-acetolactate could be made from glucose or lactose in dairy waste, respectively. The whole-cell biocatalyst was robust and fully active in crude fermentation broth containing pyruvate.

**Conclusions:**

An efficient approach for converting sugar into α-acetolactate, via pyruvate, was developed and tested successfully. Due to the anaerobic conditions used for the biotransformation, little diacetyl was generated, and this allowed for efficient biotransformation of pyruvate into α-acetolactate, with the highest titers reported to date. The use of a two-step procedure for producing α-acetolactate, where non-toxic pyruvate first is formed, and subsequently converted into α-acetolactate, also simplified the process optimization. We conclude that whole cell catalysis is suitable for converting lactose in dairy waste into α-acetolactate, which favors resource utilization.

## Background

Diacetyl, a generally recognized as safe (GRAS) compound with a pleasant buttery flavor, is widely used in the food industry [1, 2]. Diacetyl can be made by chemical or biological means. The chemical approach involves high temperature treatment of sugarcane bagasse under oxygen limiting conditions, and through a series of radical reactions diacetyl is formed [3]. Despite the harsh conditions used, the diacetyl recovered in this way is classified as natural.

**Fig. 1:**
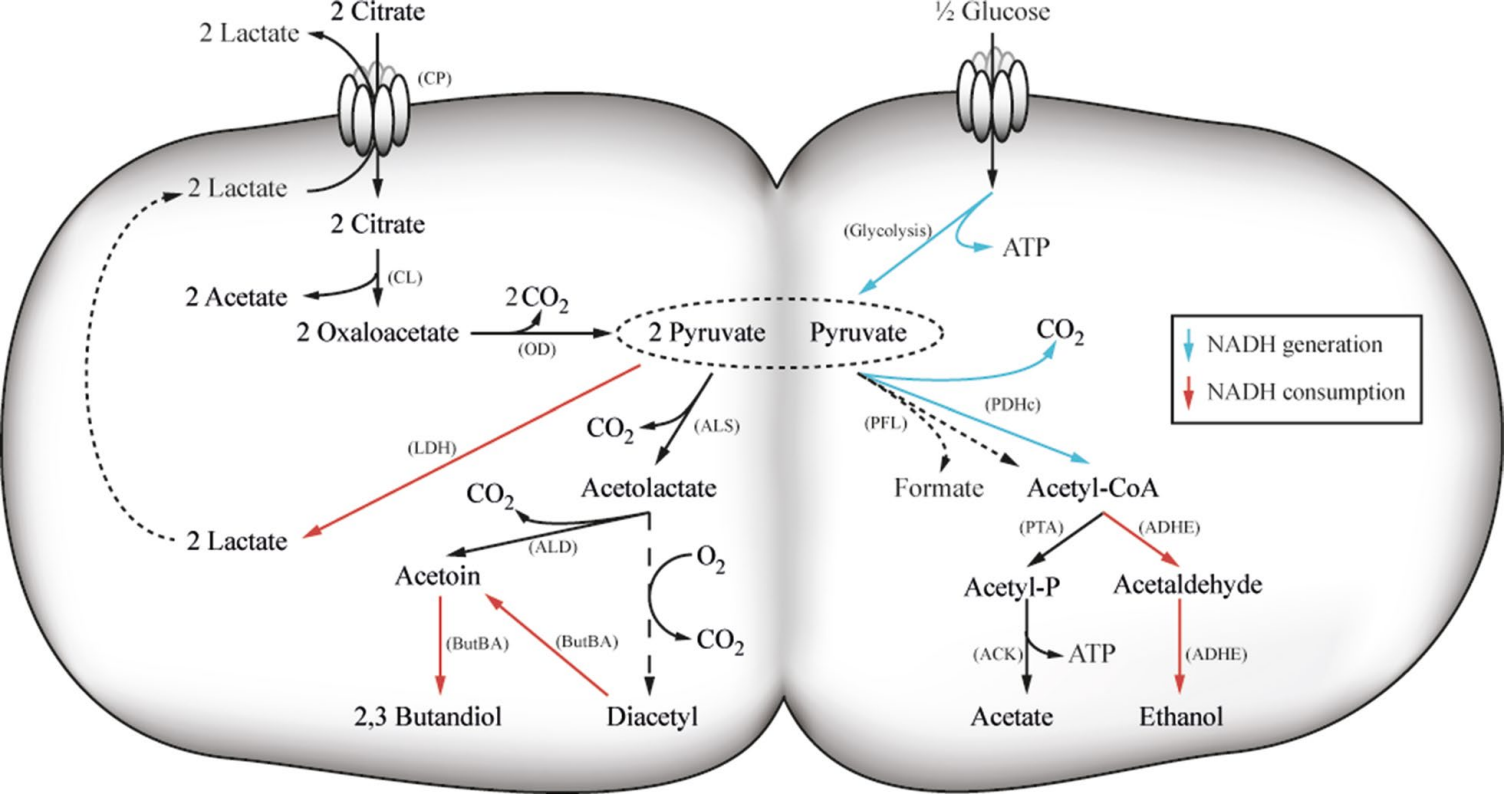
Central metabolism of *L. lactis* subsp. *lactis* biovar *diacetylactis*. Abbreviations for enzymes are: CP: citrate permease (CitP); CL: Citrate lyase; OD: Oxaloacetate decarboxylase; ALS: α-Acetolactate synthase; ALD: α-Acetolactate decarboxylase; ButBA: Butanediol dehydrogenase; LDH: Lactate dehydrogenase; PFL: Pyruvate-formate lyase; PDHc: Pyruvate dehydrogenase complex; PTA: Phosphotransacetylase; ACK: Acetate kinase; ADHE: Alcohol dehydrogenase. The NADH generating reactions are illustrated in blue arrows, while the NADH consuming ones are shown in red arrows

In traditional dairy products, such as butter, diacetyl arises due to the activity of lactic acid bacteria (LAB). Different LAB, such as *Lactococcus lactis* subsp. *lactis* biovar *diacetylactis*, *Leuconostoc* spp. [4] and various *Lactobacillus* spp. [5, 6] are able to generate diacetyl, or more precisely its precursor α-acetolactate, when citric acid is present. By metabolizing citric acid, the LAB achieve a growth advantage, since acid stress is relieved to some extent [7]. In *Lactococcus lactis* subsp. *lactis* biovar *diacetylactis* citrate is transported into the cell via the citrate permease (CitP) and cleaved by citrate lyase (CL) into acetate and oxaloacetate. The latter is subsequently decarboxylated by oxaloacetate decarboxylase (OD) into pyruvate. Thus, the consumption of citrate increases the pyruvate pool, without increasing the NADH production in the cell. The excess pyruvate is subsequently consumed via the acetoin pathway, comprised of α-acetolactate synthase (ALS) and α-acetolactate decarboxylase (ALD) (Fig. 1). α-Acetolactate, the intermediate in this pathway, is inherently unstable and can either be enzymatically converted into acetoin or undergo spontaneous decarboxylation to diacetyl under aerobic condition [4, 8–11]. As a result, α-acetolactate is mainly transformed into acetoin. However, small amounts of diacetyl can also form when oxygen is present. It has been found that the presence of metal ions accelerate the chemical conversion of α-acetolactate into diacetyl, which can be used for industrial diacetyl production [4, 12]. Even though diacetyl is a very potent flavor molecule there are some advantages of using α-acetolactate instead, since diacetyl is highly volatile, which complicates its use in food products. To avoid this, α-acetolactate can be added to the products directly and diacetyl is then formed slowly when the product gets in contact with oxygen. Thereby a long-lasting taste is obtained, and high initial concentrations of diacetyl are avoided [21].

Metabolic engineering has been used to improve diacetyl formation [12–19]. Hugenholtz et al. overexpressed NADH-oxidase (NOX) in an ALD deficient strain and managed to generate 5.7 mM α-acetolactate and 1.6 mM diacetyl using resting cells [14]. We have previously reported the highest diacetyl titer ever achieved for lactic acid bacteria, 95 mM, which was attained using a metabolically rewired strain of *L. lactis,* blocked in all major catabolic pathways except for the one leading to α-acetolactate and growing under respiratory conditions [12]. The α-acetolactate accumulated in the fermentation broth was subsequently converted into diacetyl using a metal ion catalyst. The chemical unstability of α-acetolactate, however, also resulted in diacetyl accumulation in the course of the fermentation, which ultimately affected growth and limited the production of α-acetolactate. Previously, it has been found that 10 mM of diacetyl completely inhibits growth of *L. lactis* [12, 14], and it was demonstrated that the cytoplasmic NOX was one of the enzymes that is hampered the most by diacetyl [14]. Diacetyl affects other enzymes as well, which may explain its strong antimicrobial activity [20]. In the earlier work, we relied on respiration to regenerate NAD^+^ and successfully generated large amounts of α-acetolactate and diacetyl [12], however, there are some drawbacks to this approach. Firstly, slow diacetyl accumulation took place during the fermentation, probably due to the presence of oxygen, which catalyzes the decomposition of α-acetolactate. Second, hemin adds additional costs to the fermentation process.

There are few reports describing the use of whole-cell biocatalysis for producing α-acetolactate from glucose or pyruvate [21, 22]. Using resting cells of an *L. lactis* strain with a deleted ALD (*aldB*) and an overexpressed NOX (*noxE*), 5.7 mM α-acetolactate was obtained [14]. In the patent literature it has been claimed that higher α-acetolactate titers can be obtained from pyruvate using whole cells or cell lysates of *L. lactis* [21, 22]. It thus appears that production of α-acetolactate by using whole-cell biocatalysis is possible, however, the existing processes do not seem to be commercially attractive due to low titers. It is however plausible that α-acetolactate production using a whole-cell catalysis process can be further improved, e.g. by optimizing the α-acetolactate synthase and by optimizing the production parameters.

**Fig. 2:**
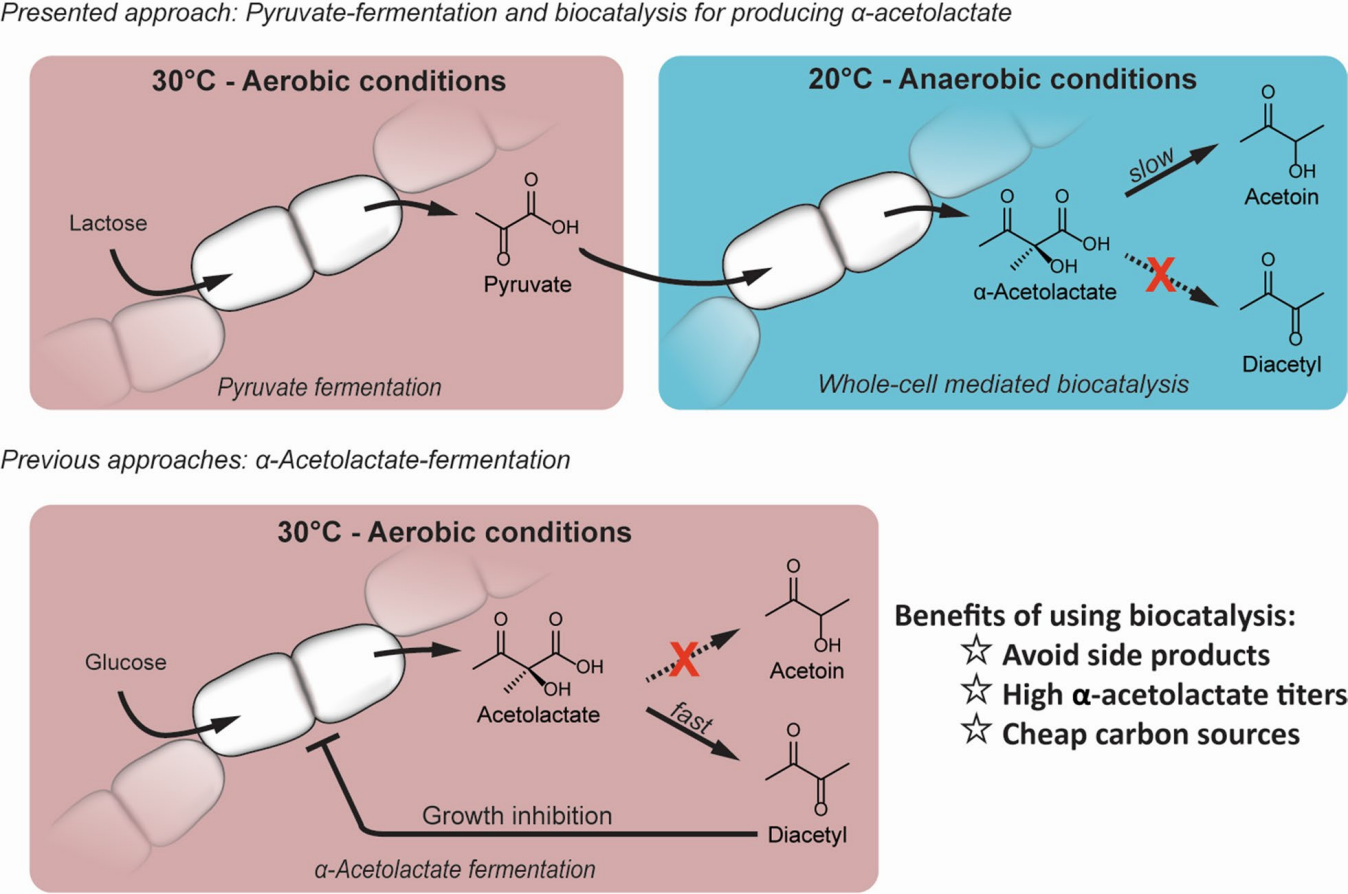
Overview of the whole cell catalyzed approach for producing α-acetolactate, which is explored in this study (upper row). Previous approaches for producing α-acetolactate directly via fermentation (lower row). Shifting the reaction environment from aerobiosis to anaerobiosis is advantageous for accumulating α-acetolactate and higher titers can be reached

Recently, we developed a pyruvate producing *L. lactis* strain, which can accumulate up to 49.7 g/l (564 mM) pyruvate from glucose or lactose under aerobic condition (Suo et al. manuscript submitted). Under anaerobic conditions, α-acetolactate or pyruvate formation directly from sugar is normally not feasible as the NADH produced during glycolysis needs to be re-oxidized, and this usually happens through either lactate formation or mixed-acid formation [23, 24]. Under aerobic conditions, however, NOX or respiration can reconstitute the NADH/NAD^+^ balance, thus allowing the cells to grow [12, 23, 24] and produce α-acetolactate, but with the drawback that inhibitory amounts of diacetyl can be formed during the fermentation. Here we explore a different strategy for producing α-acetolactate, which is based on whole cell catalyzed transformation of pyruvate (Fig. 2). We start out by constructing a suitable chassis based on *L. lactis,* then test the effectiveness of different α-acetolactate synthase enzymes and finally optimize various parameters for the cell catalyst.

## Results

### Strain construction for whole cell biocatalysis

The inherent instability of α-acetolactate, causing it to decompose to growth inhibiting diacetyl in the presence of oxygen, makes it a challenge to achieve high titers by aerated fermentation. We envisioned that using a production strategy based on whole cell based biocatalysis might be a superior approach, e.g. by using pyruvate as a substrate. Pyruvate can be obtained via fermentation using cheap carbon sources as described elsewhere (Suo et al., manuscript submitted). Diacetyl can easily be prepared from the resulting α-acetolactate by subsequent aeration, a reaction that can be further accelerated by metal-ion catalysts. In order to be efficient, the whole-cell catalyst should possess a high α-acetolactate synthase activity, and any genes encoding enzyme activities that could interfere with α-acetolactate production should be inactivated. *L. lactis* subsp. *cremoris* MG1363 is a well-characterized model strain, which we decided to use in this study. Starting from the previously described strain CS4525 (MG1363 *Δldh, ΔldhB ΔldhX Δpta ΔadhE ΔbutBA ΔaldB,* pCS4268) [12], we derived ll-cat01 (MG1363 *ΔldhB ΔldhX Δpta ΔadhE ΔbutBA ΔaldB*) by concurrent plasmid curing and selection for anaerobic growth. In this strain the chromosomal *ldh* had been restored after a recombination event with the plasmid encoded *ldh* gene. ll-cat01 satisfied the requirements to be used as a chassis for our whole cell catalyst.

### α-Acetolactate can be obtained from pyruvate using an *L. lactis* whole cell biocatalyst

**Fig. 3:**
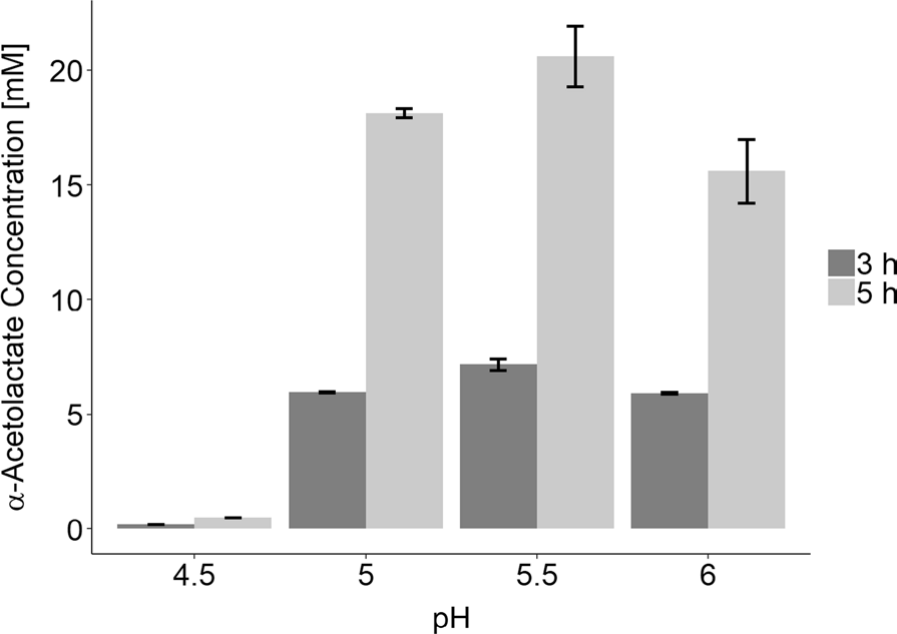
α-Acetolactate is obtained from pyruvate (500 mM) using ll-cat01 in citrate-buffered reaction solutions (pH 4.5, 5.0, 5.5 and 6.0). Concentrations after 3 h (dark) and 5 h (light) are shown

As proof of principle, we applied ll-cat01 in a solution containing 500 mM pyruvate buffered with citrate (100 mM) at different pH values in 1 ml volume. We assayed the α-acetolactate concentration during the first 5 h of the reaction using a colorimetric assay. This assay allows for determination of the acetoin concentration after converting α-acetolactate to acetoin under acidic conditions. As α-acetolactate decarboxylase had been knocked out and there is no other cofactor independent metabolic pathway, we expected pyruvate to be converted exclusively into α-acetolactate. We did observe formation of α-acetolactate, however, only at pH above 5 (Fig. 3). At pH 4.5 the catalyst appeared inactive, and no product was formed. At higher pH α-acetolactate was formed, and the maximum titer was seen after 5 h at pH 5.5 (20.6 ± 0.3 mM). Since pH increases when two equivalents of acidic pyruvate (pKa = 2.5) are converted into one equivalent of the less acidic α-acetolactate (pKa = 3.45), it is likely that higher titers could have been obtained if the pH was controlled better, and this would explain the lower activity of the cell catalyst at pH 6.0.

### Optimization of the biocatalysis process with different α-acetolactate synthases

The catalytic effeciency of α-acetolactate synthase (ALS) is likely to limit the efficiency of the biocatalysis process. Therefore, enhancing the ALS activity is important for optimizing the biocatalytic process and can be done in different ways, e.g. by overexpressing ALS or lowering the *K*_*m*_ by expressing heterologous ALS. We decided to overexpress three different ALS enzymes, the native ALS from *Lactococcus lactis* (ll-ALS), ALS from *Bacillus licheniformis* WX-02 (bl-ALS) [25] and ALS from *Enterococcus faecalis* (ef-ALS) [26], where the latter two have been reported to be robust enzymes with high affininty towards pyruvate (Additional file 1: Table S2). The three enzymes were overexpressed individually in the strain ll-cat01 using the same expression cassette, resulting in strains RD04 (ll-ALS), RD05 (bl-ALS) and RD06 (ef-ALS). As a negative control the empty plasmid (pLC0) was transformed into ll-cat01, resulting in RD03.

**Fig. 4:**
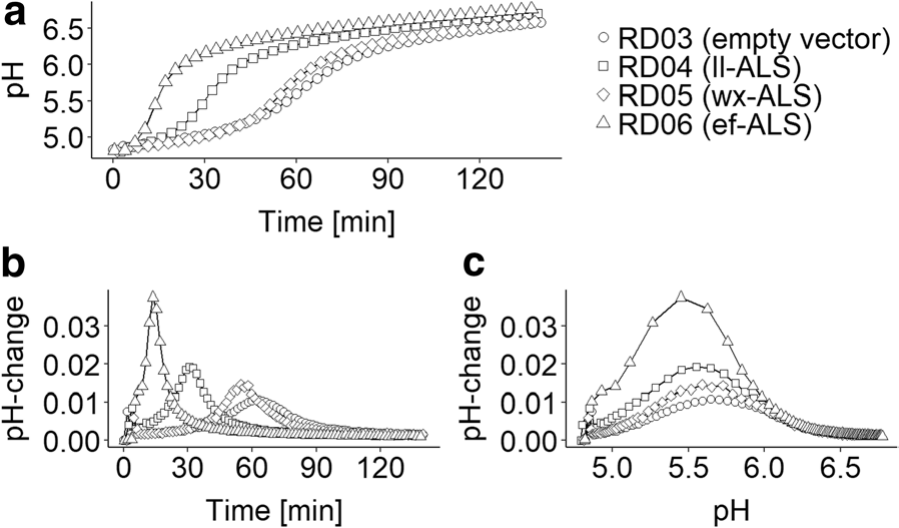
Comparison of the four strains RD03-RD06, derived from the same host ll-cat01 containing the plasmids pLC0, pLC17, pLC02, pLC01, as biocatalysts for the conversion of pyruvate to α-acetolactate. RD03-RD06 were incubated in an unbuffered solution containing 0.5 M pyruvate at pH 4.6 at OD_600_ = 1.0. RD03 contains the empty cloning vector as negative control (circles). RD04-RD06 contain plasmids over-expressing different α-acetolactate synthase enzymes from *L. lactis* (ll-ALS) (squares), *B. licheniformis* WX-02 (bl-ALS) (diamonds) and *E. faecalis* (ef-ALS) (triangels). A: pH-profile (pH against time [min]). B: delta-pH as indicator for the catalytic activity. C: delta-pH against pH. Because of the generally small errors, the error bars are not indicated here

The optimal pH for the four strains: RD03-RD06 containing the four different plasmids: pLC0, pLC17, pLC02, pLC01 was determined using pH-monitoring, which was initially established and verified with ll-cat01 using different pH at the beginning of the reaction (see Additional file 1: Figure S1). All four strains showed “S”-shapes, when monitoring pH over time, indicating functional expression of the enzymes (Fig. 4a). Moreover, the curves differed significantly from each other: While the shape for RD03 and RD05 was rather flat, the shape for RD04 and RD06 showed a very sharp increase in activity early in the experiment. The difference in shape indicates altered reaction rates, which becomes more obvious when plotting the ΔpH versus either time or pH (Fig. 4b, c). The peaks in the corresponding curves shows the time or pH when the consumption rate of pyruvate is highest and the magnitude gives an indication of the overall maximum activity. The data indicates that RD06 performs best under the given condition, followed by RD04, RD05 and RD03. This trend was confirmed in small scale biotransformations, where the pH was kept constant using a buffer (Additional file 1: Figure S2). Surprisingly, all four strains showed a very similar optimal pH between 5.5 and 5.6 (Fig. 4c). We chose RD06 for subsequent scale-up experiments because of its superior performance.

### Low temperature and high cell density are beneficial for α-acetolactate accumulation

**Fig. 5:**
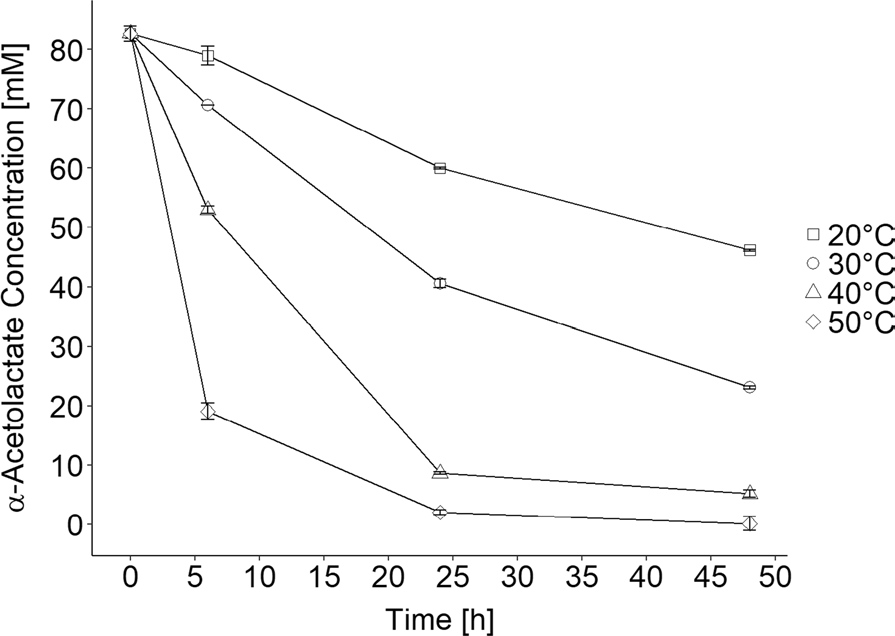
Stability of α-acetolactate at different temperatures (20 °C (squares), 30 °C (circles), 40 °C (triangles) and 50 °C (diamonds)) and in the presence of 500 mM pyruvate and 100 mM citrate at pH 5.5. α-Acetolactate decarboxylates to mainly form acetoin but also other side products

It is well known that α-acetolactate spontaneously decarboxylates in aqueous solution and forms acetoin or diacetyl depending on the absence or presence of oxygen, respectively. The process can be accelerated in acidic environments and proceeds faster at high temperature [10, 11]. To determine the optimal temperature for α-acetolactate accumulation using RD06, the stability of α-acetolactate under different conditions was evaluated. The initial concentration of α-acetolactate was determined to be approximately 83 mM. Generally, it was observed that α-acetolactate mainly decarboxylates into acetoin, but other unknown side products are formed as well, especially at high temperatures. Figure 5 shows that increasing temperatures and prolonged reaction time are suboptimal for the accumulation of α-acetolactate. At 50 °C, already after 6 h, only 19.1 ± 1.4 mM of the initial 83 mM α-acetolactate remained (23 ± 2%) and after 24 h at 40 °C and 50 °C only 8.6 ± 0.2 mM (10.4 ± 0.1%) and 2.0 ± 0.4 mM (2.5 ± 0.4%) remained, respectively. When comparing the lower temperatures, 20 °C was found to be suitable since at 30 °C after 24 h already more than half of the α-acetolactate had been degraded. We decided to apply the biocatalyst at 20 °C at laboratory scale in order to stabilize α-acetolactate and increase the biocatalyst concentration to compensate for decreased reaction rates. We found a linear correlation between cell density and α-acetolactate production (see Additional file 1: Figure S3) and concluded that higher cell densities indeed can be applied to compensate for lower reaction rates caused by lower temperatures. To validate this hypothesis, we determined the α-acetolactate formation rate [mM/min] of RD06 at different temperatures from 15 °C to 30 °C (Additional file 1: Figure S4). In this temperature range a nearly linear correlation was observed and we demonstrated that increased cell density can compensate for the lower α-acetolactate formation rate when using lower temperatures (e.g. 20 °C). Higher cell densities might also compensate for increased α-acetolactate degradation at higher temperatures when shorter reaction times are applied.

### Biotransformations under optimized conditions

**Fig. 6:**
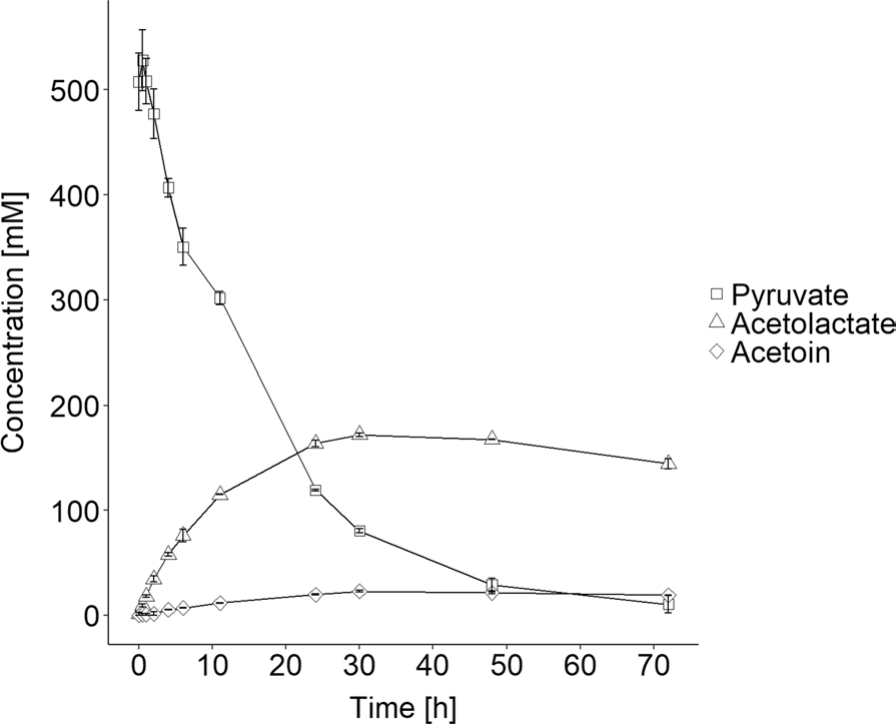
Production of α-acetolactate from pyruvate using RD06 as whole cell biocatalyst. The concentrations of pyruvate (squares), α-acetolactate (triangles) and acetoin (diamonds) are shown (mM)

As mentioned above, α-acetolactate rapidly decomposes into diacetyl in the presence of oxygen. Therefore, the following laboratory-scale biotransformations were carried out under anaerobic conditions. For the bioconversion we applied the optimal conditions established above (pH 5.5, 20 °C) and the most efficient catalyst (RD06). The consumption of pyruvate as well as the production of α-acetolactate and acetoin were followed over a period of 72 h (Fig. 6). The biotransformation reached it optimal state after 30 h, when the α-acetolactate concentration was at a maximum (172 ± 2 mM) and 472 ± 40 mM pyruvate had been consumed. We found that longer reaction times resulted in a loss of α-acetolactate because of spontaneous decarboxylation into acetoin. At the maximal α-acetolactate concentration the yield from pyruvate was 73 ± 9% (mol α-acetolactate/0.5*mol pyruvate consumed), and only smaller amounts of acetoin (23 ± 1 mM) and unknown side products were observed using HPLC (see Additional file 1: Figures S6–S8). Furthermore, during the biotransformation we observed a slight decrease in cell density, which might explain the decreased α-acetolactate formation rate in the later stages of the reaction (Additional file 1: Figure S9).

### α-Acetolactate production using a dairy side-stream as feedstock

**Fig. 7:**
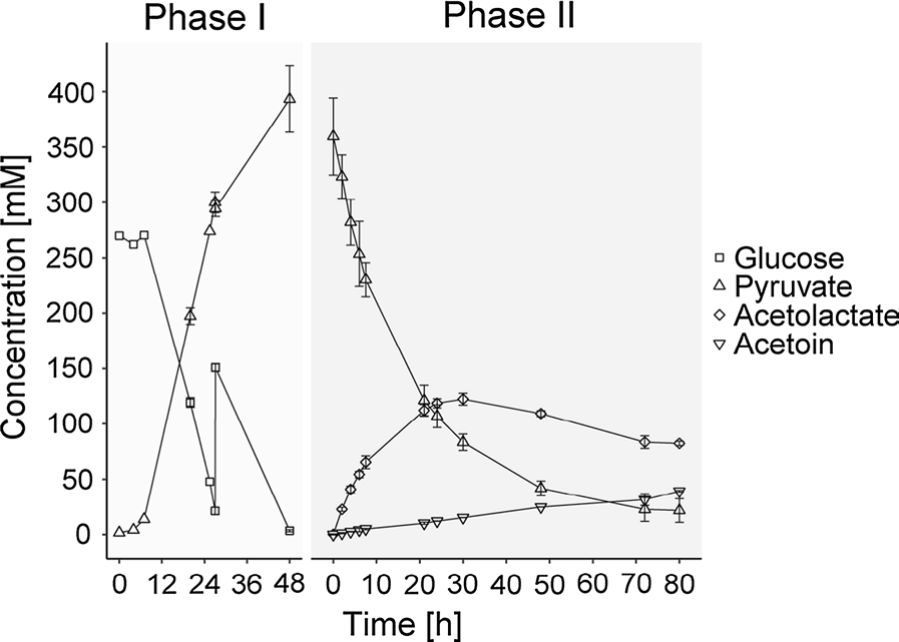
Production of α-acetolactate from glucose via pyruvate. Glucose is fermented using FS1072 in M17 broth and pyruvate is obtained (Phase I). After 74 h FS1072 was removed from the broth and RD06 was applied as whole cell biocatalyst for converting pyruvate into α-acetolactate (Phase II). The concentrations of glucose (squares), pyruvate (triangles), α-acetolactate (diamonds) and acetoin (inverted triangle) are shown [mM]. Lactate and acetate were observed at different levels after phase I (81 ± 8 mM and 38 ± 8 mM, respectively), but they remained constant during phase II and are therefore not shown

**Fig. 8:**
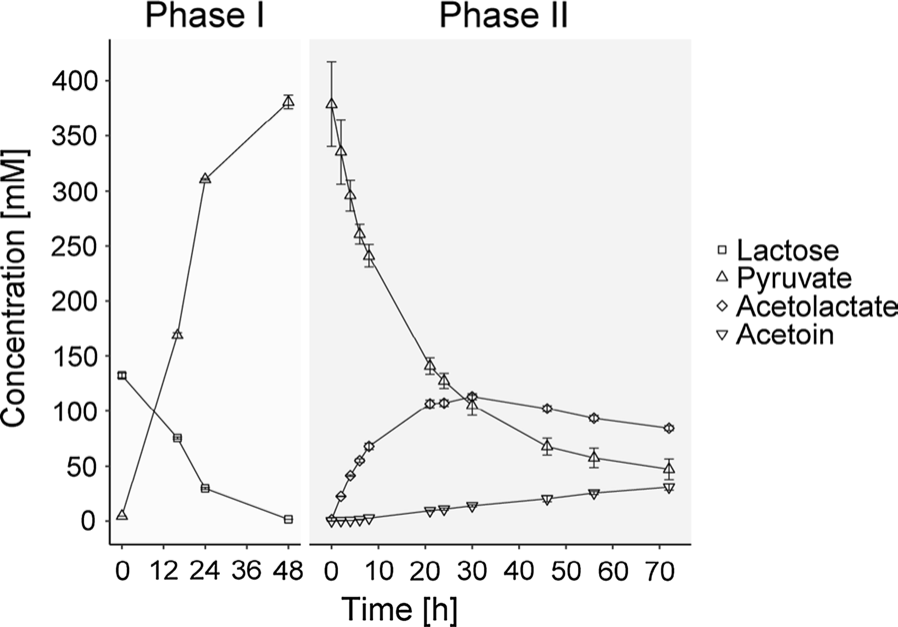
Production of α-acetolactate from lactose via pyruvate. Lactose is fermented using FS1080 in RWP and pyruvate is obtained (Phase I). After 48 h FS1080 was removed from the broth and RD06 was applied for converting pyruvate into α-acetolactate (Phase II). The concentrations of Lactose (squares), pyruvate (triangles), α-acetolactate (diamonds) and acetoin (inverted triangle) are shown (mM). Lactate and acetate were observed at different levels after phase I (68 ± 4 mM and 31 ± 4 mM, respectively), but they remained constant during phase II and are therefore not shown

We have demonstrated that our cell catalyst can transform a buffered solution of pure pyruvate efficiently into α-acetolactate, however, we do not know if the cell catalyst is active in spent fermentation broth containing pyruvate. Since we recently have generated *L. lactis* strains able to ferment either glucose or lactose into pyruvate, we decided to investigate this. The two strains FS1072 and FS1080, able to metabolize glucose or lactose respectively were used for the purpose. Two pyruvate solutions containing 393 ± 30 mM pyruvate (GM17) and 380 ± 6 mM pyruvate (residual whey permeate) were produced (Phase I, Figs. 7 and 8). The pyruvate titers obtained using these strains were somewhat lower than previously observed, but since the objective here was to demonstrate the functionality of the cell catalyst, we did not pursue optimization of the pyruvate fermentation, which is described elsewhere (Suo et al. manuscript submitted). Indeed, the cell catalyst turned out to be highly active in the presence of the various components present in the rich media used, which demonstrates the robustness of the biocatalyst. The α-acetolactate concentrations reached maxima after 30 h in phase II with 122 ± 5 mM or 113 ± 3 mM in GM17 or RWP, respectively. After 30 h, 15.2 ± 0.2 and 13.8 ± 0.9 mM acetoin had been formed in GM17 and RWP, respectively. However, in contrast to when using pure pyruvate, a nearly linear increase in acetoin concentration was observed for both reactions after 30 h. This indicates that α-acetolactate stability is compromised in GM17 and RWP, as the increase in acetoin concentration is directly ascribed to the decomposition of α-acetolactate. To summarize, α-acetolactate yield from pyruvate was 80 ± 3% (mol α-acetolactate/0.5*mol pyruvate consumed) for the reaction in GM17-broth and 76 ± 11% (mol α-acetolactate/0.5*mol pyruvate consumed) in RWP-broth. Compared to the biotransformation using pure pyruvate, the yields were similar. The overall yield from glucose or lactose were 44 ± 6% (mol α-acetolactate/mol glucose consumed) or 43 ± 14% (mol α-acetolactate/2*mol lactose consumed), which are both rather low, because of the non-optimized pyruvate fermentations.

## Discussion

We have demonstrated that biocatalysis is applicable for producing high titers of α-acetolactate from pyruvate, either pure or fermentation derived. Due to its unstable nature, it is difficult to obtain high titers of α-acetolactate by fermentation, and the best results this far have been realized using aerated conditions [12, 27]. Using anaerobic conditions in combination with a cell catalyst, in this study, we managed to reach 172 ± 2 mM of α-acetolactate.

Normally, when producing so-called starter distillates, diacetyl in fermented broth is concentrated by steam distillation to prepare a potent flavoring agent, and the remaining liquid constitutes waste. In this study, we achieved higher concentrations of α-acetolactate, which can be quantitatively converted to diacetyl, e.g. using Fe^3+^-ions and the obtained product potentially could be added directly to food products.

We find that, for all cell catalysts tested, the optimal pH is around 5.5. This is a little surprising since slightly different pH-optima have been reported previously for the different ALS enzymes used (Additional file 1: Table S2). It is likely that the low K_m_ of ef-ALS is responsible for the superior performance of RD06. As all three enzymes were overexpressed from the same expression cassette, similar expression values are expected, but potentially bl-ALS is expressed less efficiently due to the lower phylogenetic relationship between lactococci and bacilli explaining the inferior performance of RD05. In future work, metabolic, protein or process engineering is likely to further enhance the efficiency and applicability of the presented process. As indicated above, biotransformation at higher temperatures such as 30 °C with increased cell density (e.g. OD_600_ = 10.0) would accelerate the conversion considerably. However, at the same time, α-acetolactate would be less stable and side products would form more rapidly. Nevertheless, a shorter biotransformation under those conditions might be desired in some industrial settings. For other cell catalysts, permeabilization was beneficial for improving efficiency [28, 29]. Unfortunately, our attempts to permeabilize the whole-cell biocatalyst using ethanol resulted in decreased reaction rates and reduced α-acetolactate yields (data not shown). It is possible that ALS requires a reduced environment for full functionality as present within intact cells. Another aspect of commercialization is the reusability of the cells, because it is expensive to produce biomass. Reusability would ease large scale applications of the presented technology. Surprisingly, when cells, which had been used for a biotransformation in pyruvate solution, were reused under the same reaction conditions, the formation of a different product was observed, which we have not identified yet. Further attempts for reusing the cells are ongoing.

## Conclusion

In this study, we have demonstrated the production of α-acetolactate from pyruvate (172 ± 2 mM) and a dairy derived waste stream (113 ± 3 mM). The reported product titers are exceeding previously published results. In our experiments, we used bioreactors for the biotransformation of pyruvate into α-acetolactate, which makes a subsequent upscaling easier because the conditions can be more directly transferred to larger vessels. The presented process further provides a solution for the recycling of a dairy waste stream into a desired food ingredient.

## Methods

### Bacterial strains and DNA techniques

ll-cat01 (MG1363 *ΔldhB ΔldhX Δpta ΔadhE ΔbutBA ΔaldB*) is a plasmid free strain derived from *L. lactis* MG1363 [30]. Gene deletions were achieved using the method developed by Solem et al. [31]. The vector pLC0 is a size-reduced derivative of pTD6 [32] lacking the *gusA* gene. The α-acetolactate-synthase genes from *Enterococcus faecalis* ATCC 29212 (ef-ALS), *Bacillus licheniformis* WX-02 (bl-ALS) [25], and *Lactococcus lactis* MG1363 (ll-ALS) were cloned into pLC0 resulting in the plasmids pLC01, pLC02, and pLC17, respectively. To overexpress these genes, they were put under the control of the strong constitutive promoter P8 [33].

FS1072 (Table 1) was constructed based on the strain CS4363 [32] by deleting the *als* gene. To make the strain able to use lactose as carbon source, the plasmid pLP712 was extracted from the strain NCDO712 and transformed into the strain FS1072, resulting in the strain FS1080 (Table 1) (Suo et al. manuscript submitted).

**Table 1:** Bacterial strains and plasmids used in this study

Designation	Genotype or description	Reference
*L. lactis* strains		
MG1363	Plasmid free *L. lactis* strain	[30]
FS1072	MG1363 *△*^*3*^*ldh △pta △adhE △als*	(Suo et al.)
FS1080	FS1072 containing the pLP712 plasmid	(Suo et al.)
ll-cat01	MG1363 *ΔldhB ΔldhX Δpta ΔadhE ΔbutBA ΔaldB*	This work
RD03	pLC0 in ll-cat01	This work
RD04	pLC17 in ll-cat01	This work
RD05	pLC02 in ll-cat01	This work
RD06	pLC01 in ll-cat01	This work
Plasmids		
pLP712	Plasmid from *L. lactis* NCDO712 encoding lactose utilization gene custer	[30]
pLC0	pTD6 derived plasmid	This work
pLC01	pLC0 inserted with the *als* gene from *Enterococcus faecalis* ATCC 29212 (ef-ALS)	This work
pLC02	pLC0 inserted with the *als* gene from *Bacillus licheniformis* WX-02 (bl-ALS)	This work
pLC17	pLC0 inserted with the *als* gene from *Lactococcus lactis* MG1363 (ll-ALS)	This work

### Construction of the plasmids pLC0, pLC01, pLC02, and pLC17

PCR products *pLC0*-*L* amplified from the plasmid pTD6 with the primers *pTD*-*CF1* and *pTD*-*CR1* (see Additional file 1: Table S1) were circularized using T4 DNA ligase after phosphorylation to construct the plasmid pLC0. The DNA fragment *P8*-*F* containing the P8 promoter [33] was obtained by PCR using primers *KF147*-*P8F* and *KF147*-*P8R* (see Additional file 1: Table S1) and genomic DNA of *Lactococcus lactis* subsp. *lactis* KF147 as template. The DNA fragment *efALS*-*F* was amplified from *Enterococcus faecalis* ATCC 29212 (purchased from German Collection of Microorganisms and Cell Cultures GmbH, DSM No.: 2570) with the primers *ALS*-*EF*-*CF1* and *ALS*-*EF*-*CR1* by PCR (Additional file 1: Table S1). The DNA fragment *wxALS*-*F* was obtained by PCR, using the primer pair *WX02*-*ALS*-*F/WX02*-*ALS*-*R* (see Additional file 1: Table S1) and synthetic DNA as template (see Additional file 1). The DNA fragment *llALS*-*F* was amplified from genomic DNA of *Lactococcus lactis* MG1363 using primers *ALS*-*Ll*-*CF1* and *ALS*-*Ll*-*CR1* (see Additional file 1: Table S1). The fragments P8-F and *efALS*-F were inserted the vector *pLC0*-*L* simultaneously by using the In-Fusion^®^ HD Cloning Kit (https://www.takarabio.com) to generate the plasmid pLC01. The plasmids pLC02 and pLC17 were constructed in the same way but with the fragments *wxALS*-*F* and *llALS*-*F*, respectively.

### Fermentation for producing pyruvate and broth treatment

Pyruvate was produced from glucose or lactose contained in Residual Whey Permeate (RWP) as described by Suo et al. (manuscript submitted) with minor modifications. Fermentations were carried out in Biostat A bioreactors equipped with 1-L vessels. For glucose fermentations, M17 medium was used. The seed culture (20 ml) was inoculated 1/100 from an overnight culture in a shake flask using M17 medium containing 1% (w/v) glucose and incubated with strong shaking at 30 °C for 16 h. Subsequently, the seed culture was used to inoculate 800 ml M17-medium 1/100. Initially, 5% glucose was added to the medium and another 2% was added subsequently when the sugar concentration decreased below 3% (after approximately 24 h). Temperature and pH were kept constant at 30 °C and pH 7.0, respectively. The fermenters were aerated using air at 1000 ccm/min and stirring at 500 rpm. Glucose was fully depleted after 72 h and the medium harvested. When fermenting RWP, the same conditions were applied. RWP was diluted twofold to lower lactose concentration to 5–6% lactose, and was supplemented with 2% yeast extract (autoclaved separately). The fermentation was terminated after 48 h when lactose was depleted. Cells were removed from the fermentation broth by centrifugation (10,000×*g*, 10 min) and the pH of the resulting pyruvate-rich broth was adjusted to 5.5 using 1 M HCl.

### Whole-cell biocatalyst preparation and biotransformation

Small-scale reactions were carried out in 1 ml scale using 1.5 ml reaction tubes. Cells were grown in GM17 broth, containing tetracycline (5 µg/ml) when required, at 30 °C for 16 h overnight. The cells were harvested by centrifugation, washed once with cold Tris/NaCl-buffer (100 mM/150 mM, pH 6.5) and suspended in H_2_O. The optical density (OD_600_) was determined in duplicate and set to approximately OD_600_ = 50. During harvesting, cells were kept on ice. For starting the reactions, the cell suspensions were centrifuged and cells were resuspended in solutions containing pyruvate, either pure or contained in fermentation broth. The cell suspensions were incubated at the set-point temperatures in ThermoMixer with shaking. Negative control reactions were carried out in the same way but without any pyruvate.

For laboratory scale biotransformations, cells were grown anaerobically in 5 ml GM17 broth at 30 °C for overnight. Subsequently, 500 ml cultures in 1-L shake flasks were inoculated 1:1000. After grown for 14–16 h at 30 °C with shaking, cells were harvested as described above. Laboratory scale biotransformation were carried out in 1-L fermenters (Sartorius “Biostat-A” equipped with a “UniVessel Glass”). The reaction vessel was filled with 350 ml 0.5 M pyruvate solution or pyruvate containing fermentation broth. Before adding the cells, the reaction solution was flushed with N_2_ and cooled to 20 °C. During the reaction, the pH was kept constant at 5.5 using 1 M HCl. The acid consumption was recorded during the process (see Additional file 1: Figure S5) and samples were taken regularly for analysis. Dilution factors were applied to the results.

### Optimization of the reaction conditions

Determination of the optimal pH for the reaction and comparison of different whole-cell biocatalysts was carried out using an iCinac instrument. For this purpose, 10 ml of non-buffered pyruvate solution (0.5 M) was prepared and the pH was set to 4.6, which is the lowest pH allowing α-acetolactate synthase reaction. This solution was equipped with a pH-electrode and after stabilization of pH and temperature cells were added to reach a final OD_600_ 2.5. Immediately after addition of the cells, the pH recording was started. The resulting pH-shift allowed the determination of the optimal pH for the reaction and comparison of different whole-cell biocatalysts.

For determining the optimal temperature for the biotransformations, α-acetolactate stability was investigated. In this case α-acetolactate was obtained by hydrolyzing ethyl-2-acetoxy-2-methylacetoacetate using an equimolar amount of NaOH and incubating at room temperature for 1 h. The resulting hydrolysate was added to a solution containing 500 mM pyruvate and 100 mM citrate at pH 5.5. The mixture was incubated at 20 °C, 30 °C, 40 °C and 50 °C, and the concentration of α-acetolactate and side products was followed over time.

### Analytical methods

Cell growth was monitored by measuring the optical density at 600 nm (OD_600_). Determination of glucose, lactose, pyruvate, lactate and acetoin was carried out by HPLC equipped with the Aminex HPX-87H column (Bio-Rad, Hercules, USA) and RI detector. Additionally, pyruvate was determined by UV detector at 210 nm. The column oven temperature was set at 60 °C and 5 mM H_2_SO_4_ was used as the mobile phase at a flow rate of 0.5 ml/min. The α-acetolactate was quantified after conversion to acetoin, which was subsequently analyzed by HPLC, whereby the conditions for the conversion were adapted from [10]. Briefly, the samples were diluted three-fold in water (reference sample) and threefold in 0.5 M HCl (treated sample). Subsequently, the treated sample was incubated 30 min at 44 °C to allow decarboxylation of α-acetolactate to acetoin, while the reference sample was stored at 4 °C in order to prevent this reaction. Finally, both samples were analyzed using HPLC. The α-acetolactate concentration was obtained by subtracting the acetoin concentration of the reference sample from the acetoin concentration of the treated sample and dividing by the factor 0.62 [10, 34].

Alternatively, acetoin and α-acetolactate concentrations were determined using a colorimetric assay. 50 µl of reaction mixtures were added in a 96-well micro-titer-plate (MTP). 5 µl 2 M H_2_SO_4_ were added to the mixtures for decarboxylation of α-acetolactate to acetoin, which was completed by incubation for 15 min at 60 °C. Subsequently, 45 µl 0.5% creatine in H_2_0 and 45 µl 5% 1-Naphthol in 2 M NaOH were added and mixed by pipetting several times. Color formation was completed by first incubating for 15 min at 60 °C and then 15 min at 20 °C. Finally, the absorbance at 530 nm was measured using a plate reader. As a blank value, negative control reactions without pyruvate were used. A standard curve was measured each time using acetoin (0–1 mM) dissolved in the respective reaction solution. The procedures above are based on the method developed by W. Westerfeld for detecting acetoin and were adapted and modified as previously described [26, 35, 36].

## Supplementary information

**Additional file 1: Table S1.** Primers and gBlock sequence (bl-ALS). **Table S2**. Catalytic properties of used ALS enzymes. **Figures S1-S9**. Supplementary results.

## Data Availability

All data generated in this study are included in this article.
