# Supplementary material for: Efficient production of α-acetolactate by whole cell catalytic transformation of fermentation-derived pyruvate
Source: Microb Cell Fact. 2019 Dec 29;18:217. doi: 10.1186/s12934-019-1271-1 (PMC6936138; doi:10.1186/s12934-019-1271-1)
Supplement: Supplementary file 1 — Additional file 1: Table S1. Primers and gBlock sequence (bl-ALS). Table S2. Catalytic properties of used ALS enzymes. Figures S1-S9. Supplementary results. [file 12934_2019_1271_MOESM1_ESM.docx]

**Additional file 1**

**Supplementary Information**

Table S 1 Primers und gBlock sequence (bl-ALS) used in this study

| Designation | Sequence (5’🡪3’) |
| --- | --- |
| pTD‑CF1 | TAGTAGCTAGCTAGTAAAAAAAAGAACCCGAGTGG |
| pTD‑CR1 | GCATGCGGCCGCATATGCCATG |
| KF147‑P8F1 | TGGCATATGCGGCCGCATGCgataaaatttctaatgatttttttaggacaatTatttctcataaaaagcagattttag |
| KF147‑P8R | CATTATATCTCTCCATTTCTGCTGC |
| ALS‑EF‑CF1 | GCAGAAATGGAGAGATATAAtgagtaaaaaaggatcagatatcatagtagaaag |
| ALS‑EF‑CR1 | TTTTTACTAGCTAGCTACTActaataaagttgatctggtaacaatgttttacc |
| WX02‑ALS‑F | AGCAGAAATGGAGAGATATAATGAATAATGTAGCCGCTAAAAATGAAACTC |
| WX02‑ALS‑R | TTTTTACTAGCTAGCTACTATCAAGATTGCTTAGAGGCTTCTTTAT |
| ALS‑Ll‑CF1 | GCAGAAATGGAGAGATATAatgtctgagaaacaatttggggcg |
| ALS‑Ll‑CR1 | TTTTTACTAGCTAGCTACTAtcagtaaaattcttctggcaataatttttctgc |
| gBlock sequence of Bacillus licheniformis WX‑2 ALS (bl-ALS) | TGTAGCTGAATGAAATTGAAAGGAATTAAATTGAATAATGTAGCCGCTAAAAATGAAACTCTTACTGTAAGAGGAGCAGAGCTTGTGGTGGATAGTCTCATTCAGCAAGGTGTCACTCATGTTTTCGGTATTCCGGGAGCGAAAATCGATGCGGTGTTTGACGTATTGAAAGACAAGGGGCCTGAATTGATCGTTTGCCGTCACGAGCAGAATGCAGCATTTATGGCGGCGGCAGTCGGACGATTGACTGGAAAGCCCGGTGTTTGCCTGGTGACTTCAGGTCCGGGAGCGTCTAATTTAGCGACCGGTCTTGTAACAGCCAATACAGAAGGAGATCCGGTTGTTGCCCTGGCGGGTGCTGTAAAAAGAGCGGATCGTCTCAAAAAAACTCATCAATCGATGGATAATGCGGCGTTGTTTCAGCCGATTACGAAATATAGCGCAGAAGTGGAAGATGCGAACAACATACCTGAGGCTGTAACCAATGCATTCAGAGCGGCGGCTTCTGGACAGGCTGGCGCAGCGTTTCTCAGCTTTCCGCAGGACGTTACGGCCGGTCCGGCAACTGCCAAGCCGGTGAAAACCATGCCGGCGCCGAAGCTGGGCGCGGCTTCGGACGAACAAATCAGCGCGGCCATCGCCAAAATTCACAATGCGAACCTTCCTGTCGTGCTTGTCGGGATGAAAGGCGGAAGACCTGAAGCGATTGAAGCGGTTCGGCGTCTGCTAAGGAAAGTGAAACTGCCGTTTGTTGAAACATACCAAGCAGCGGGTACGCTGTCTCACGATTTGGAAGACCAGTACTTCGGCCGGATCGGACTATTCCGCAATCAGCCCGGAGACATGCTATTGGAAAAAGCGGATGTCGTTTTGACGGTCGGCTATGATCCGATTGAATACGATCCGGTCTTTTGGAATGGAAAAGGCGAACGAAGCGTGATTCATCTTGACGAAATACAAGCCGATATCGATCATGACTATCAGCCCGAAATCGAGTTGATCGGCGATATCGCAGAGACGTTAAACCATATTGAACATGATTCTCTGCCGGTTTCCATCGACGAGTCCTTTGCACCTGTGCTTGATTACTTGAAGAAAGCACTGGAGGAGCAAAGCGAACCTCCTAAAGAAACAAAAACTGATCTTGTTCATCCGCTGCAAATCGTTCGCGATTTGCGTGAACTGCTGAGCGATGACATAACGGTGACTTGCGACATCGGCTCCCATGCGATTTGGATGTCTAGATATTTCCGCACCTACCGTCCGCATGGACTGCTGATTTCCAACGGCATGCAGACGCTTGGGGTGGCTTTGCCGTGGGCGATTGCAGCAACGCTGGTCAATCCGGGACAGAAAGTCGTGTCTGTTTCCGGGGACGGGGGCTTCCTGTTCTCCGCGATGGAGCTTGAGACAGCGGTCAGATTAAAAGCGCCGATCGTTCACATTGTCTGGAATGACAGCACATACGATATGGTTGCGTTCCAGCAGGAGATGAAATACAAGCGGACGTCCGGAGTCGACTTCGGCGGAATCGATATTGTAAAATATGCTGAAAGCTTTGGTGCAAAAGGTTTAAGGGTCAATTCACCTGACGAATTGGCAGAGGTGCTTAAAGCTGGTCTTGATGCAGAGGGGCCTGTTGTCATTGATATTCCGGTCGATTACAGCGACAACATTCATTTGGCCGATCAGCGTTTTCCAAAAAAATTTGAGGAACATTTTAATAAAGAAGCCTCTAAGCAATCTTGATAAAAAAAGAACCCGAGTGGGTTCTTTTTTATTTTTTAAGTCGACCTGCAGGCATGCAAGCTTGCACT |

Table S 2 Comparison of the catalytic properties of different catabolic α‑acetolactate synthases.

| **Strain** | **Specific Activity (U/mg)** | **K_m_ for Pyruvate (mM)** | **K_cat_ (1/s)** | **Optimum pH** | **References** |
| --- | --- | --- | --- | --- | --- |
| *B. licheniformis*WX‑02 | 35.4 | 3.96 | 514 | 6.5 | (26) |
| *L. lactis* | 103^†^ | 50 | NR | 6.5‑7.0 | (28) |
| *E. faecalis* | 5.36 | 1.37 | NR | 6.8 | (27) |

†: The method for purifying the enzyme differs in comparison to the other two enzymes. NR: not reported

**Supplementary Results**


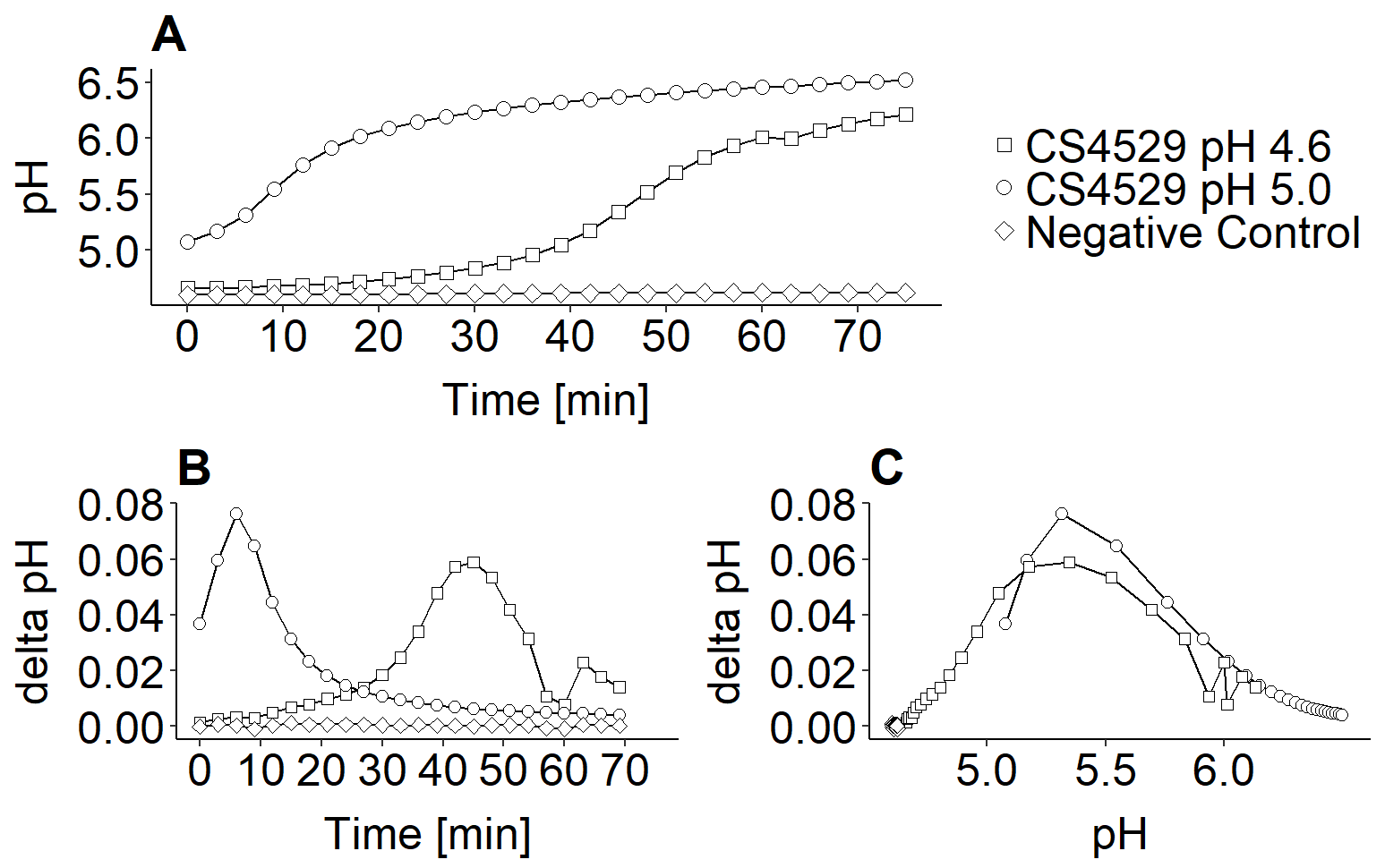


Figure S 1 ll-cat01 was incubated in an unbuffered solution containing 0.5 M pyruvate at pH 4.6 and pH 5.0 at OD_600_ = 2.5. As negative control, ll-cat01 was incubated in a solution without pyruvate. A: pH‑profile (pH against time [min]). B: delta‑pH as indicator for the catalytic activity over time. C: delta‑pH against pH.


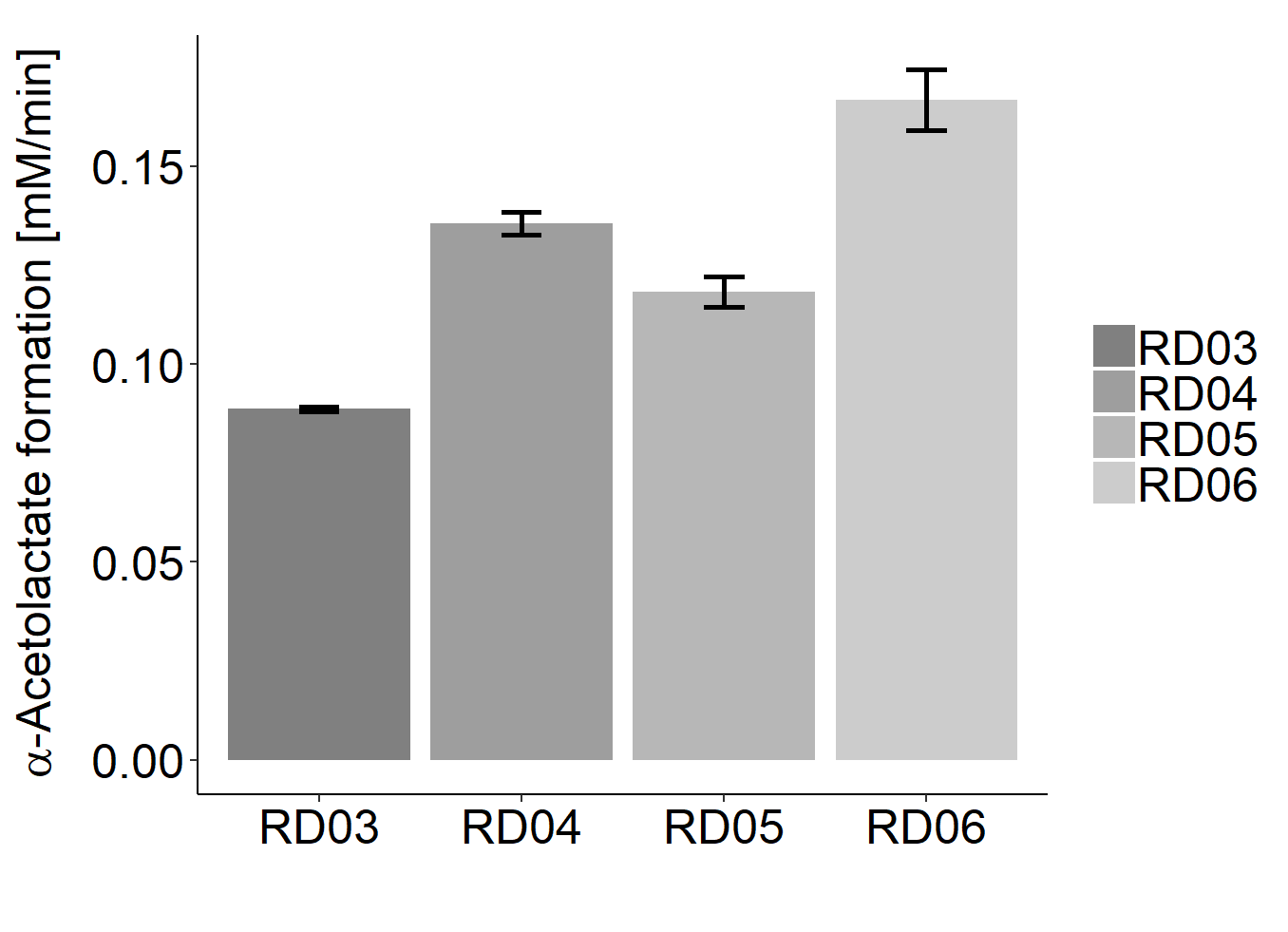


Figure S 2 Comparison of RD03 – RD06 using small-scale biotransformations. The α-acetolactate formation rate [mM/min] was determined at constant pH. The biocatalysts were applied at a cell density corresponding to OD_600_ = 2.5 at 20°C.


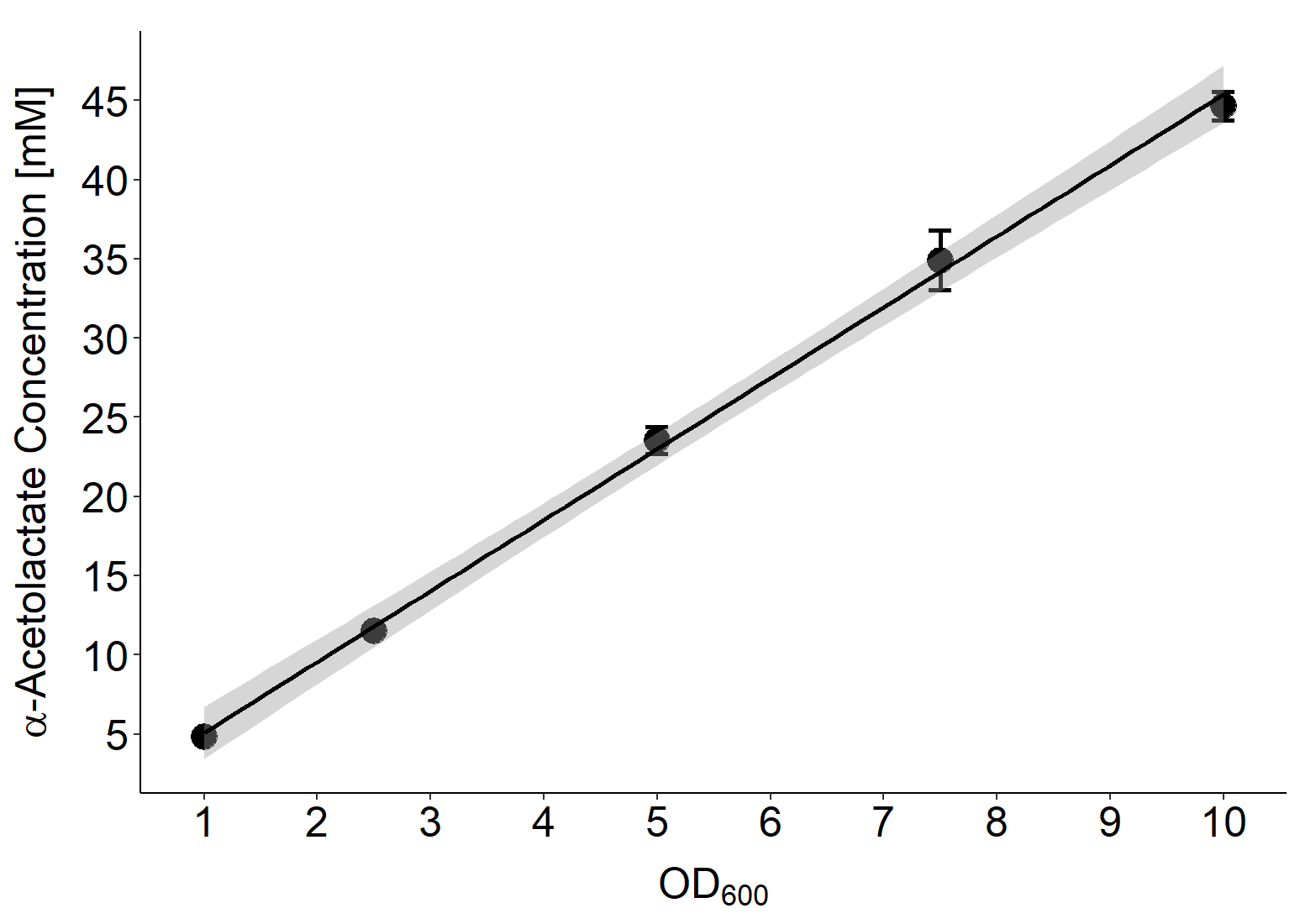


Figure S 3 Linear correlation between OD_600_ and α‑acetolactate concetration produced by RD06 after 2 h in 500 mM pyruvate buffered with 100 mM citrate at pH 5.5. The regression follows: y = 4.478 * x + 0.598 (R^2^ = 0.9986).


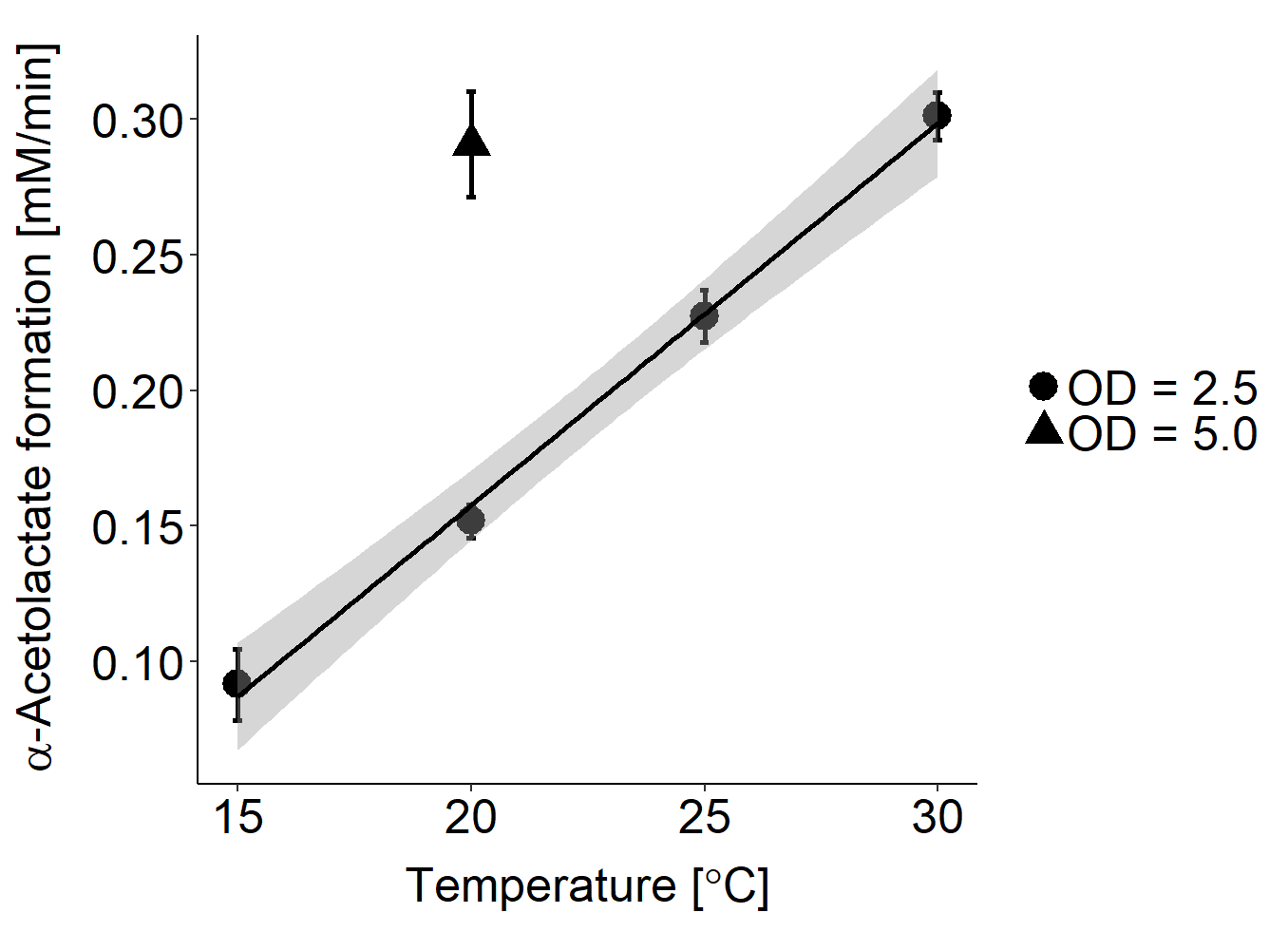


Figure S 4 α-Acetolactate formation rate [mM/min] for RD06 at different temperatures and cell densities (OD = 2.5 (circles) and OD = 5.0 (triangles). A nearly linear correlation between temperature and formation rate was observed. Increased cell density can compensate for decreased activity at lower temperatures as indicated at 20°C.


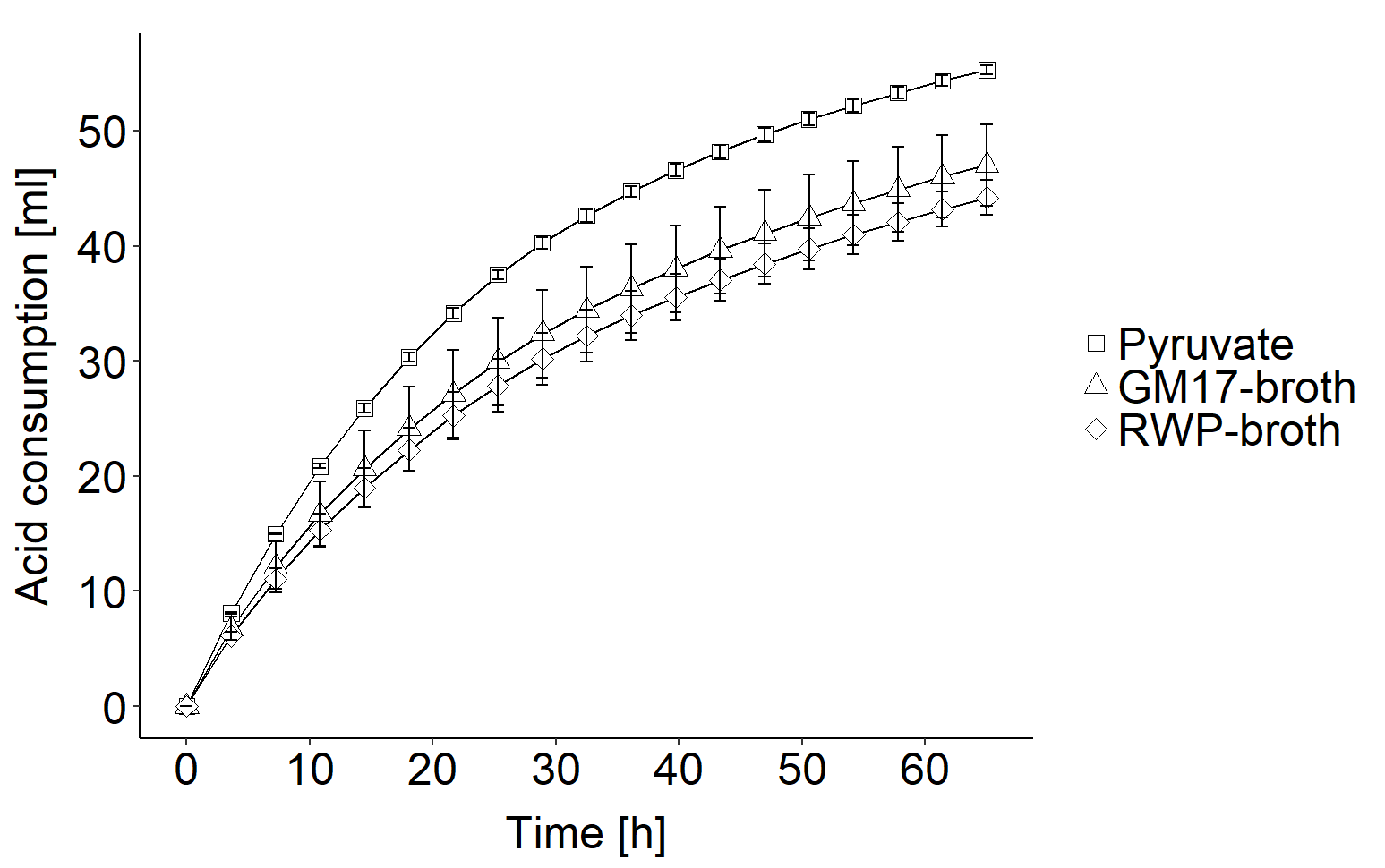


Figure S 5 Acid consumption during the laboratory scale biotransformation. Data is the average of two independent experiments.


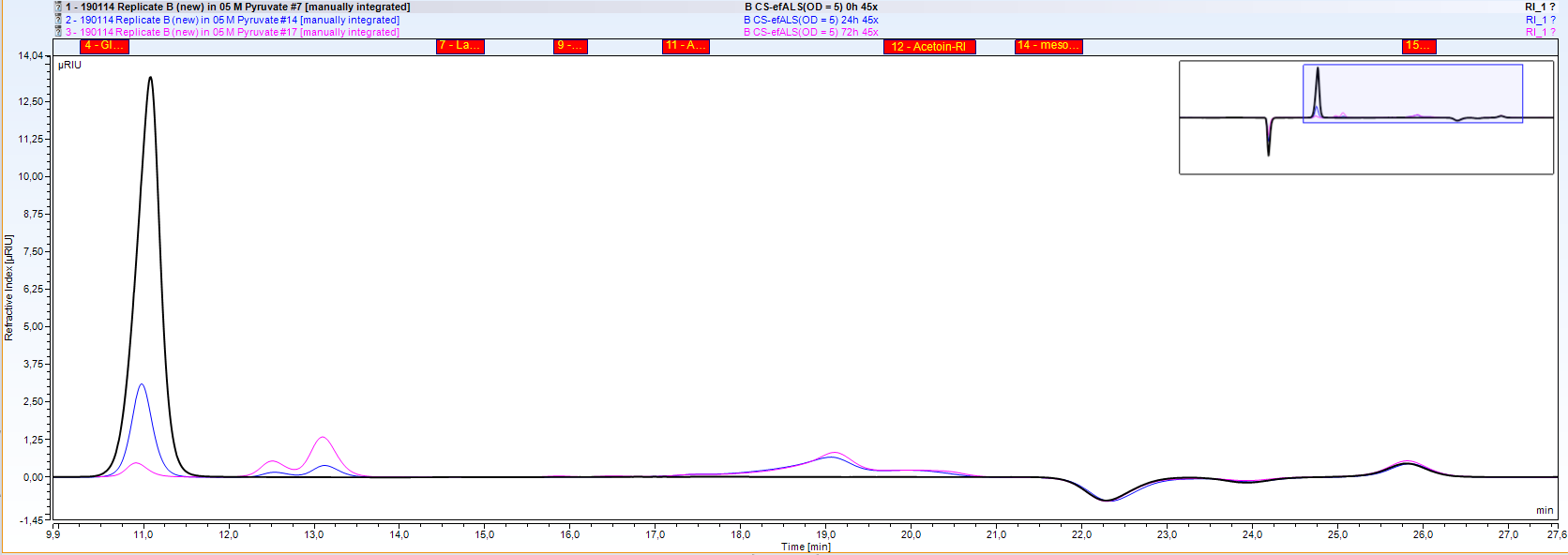


Figure S 6 HPLC‑spectrum of reactions of RD06 in pyruvate solution with 45x dilution. Results after 0 h (black), 24 h (blue) and 72 h (pink) are shown. Retention times are as follows: Pyruvate: 10.8 min, α‑Acetolactate: 19.0 min, Acetoin: 20.7 min, Unknown Side product: 13.1 min.


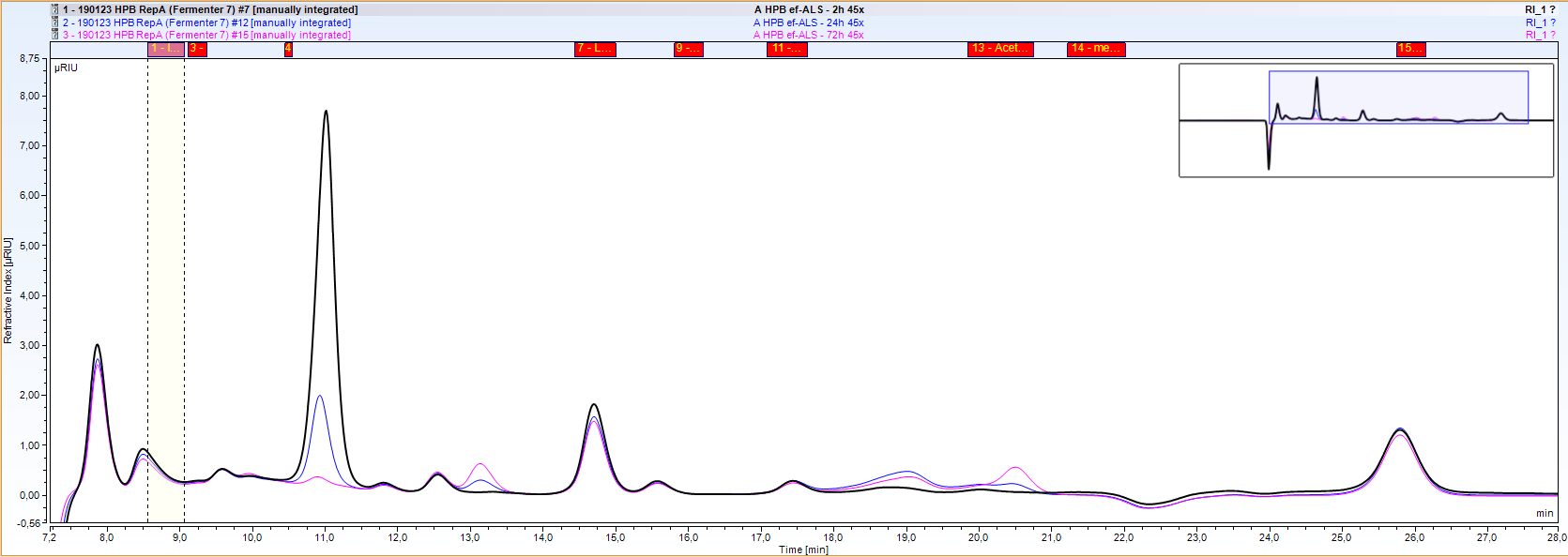


Figure S 7 HPLC‑spectrum of reactions of RD06 in pyruvate‑containing GM17‑broth with 45x dilution. Results after 0 h (black), 24 h (blue) and 72 h (pink) are shown. Relevant retention times are as follows: Pyruvate: 10.7 min, α‑Acetolactate: 18.9 min, Acetoin: 20.5 min, Unknown Side product: 13.1 min.


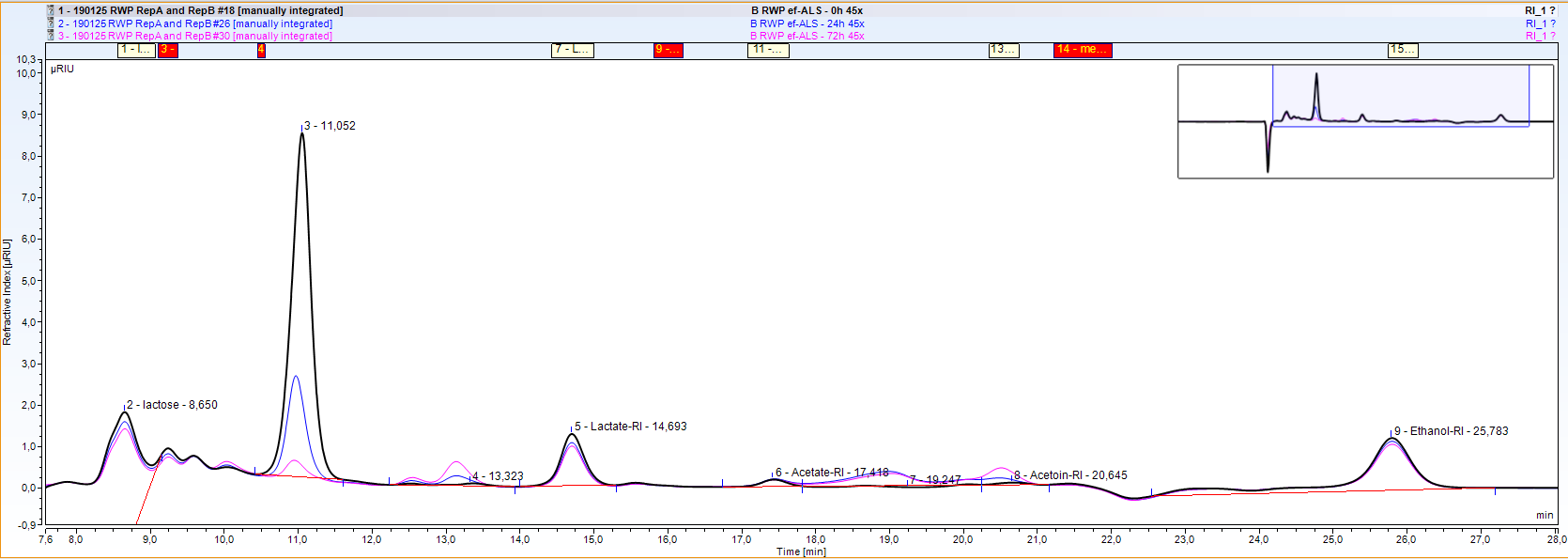


Figure S 8 HPLC‑spectrum of reactions of RD06 in pyruvate‑containing RWP with 45x dilution. Results after 0 h (black), 24 h (blue) and 72 h (pink) are shown. Relevant retention times are as follows: Pyruvate: 10.7 min, α-Acetolactate: 19.0 min, Acetoin: 20.5 min, Unknown Side product: 13.2 min.


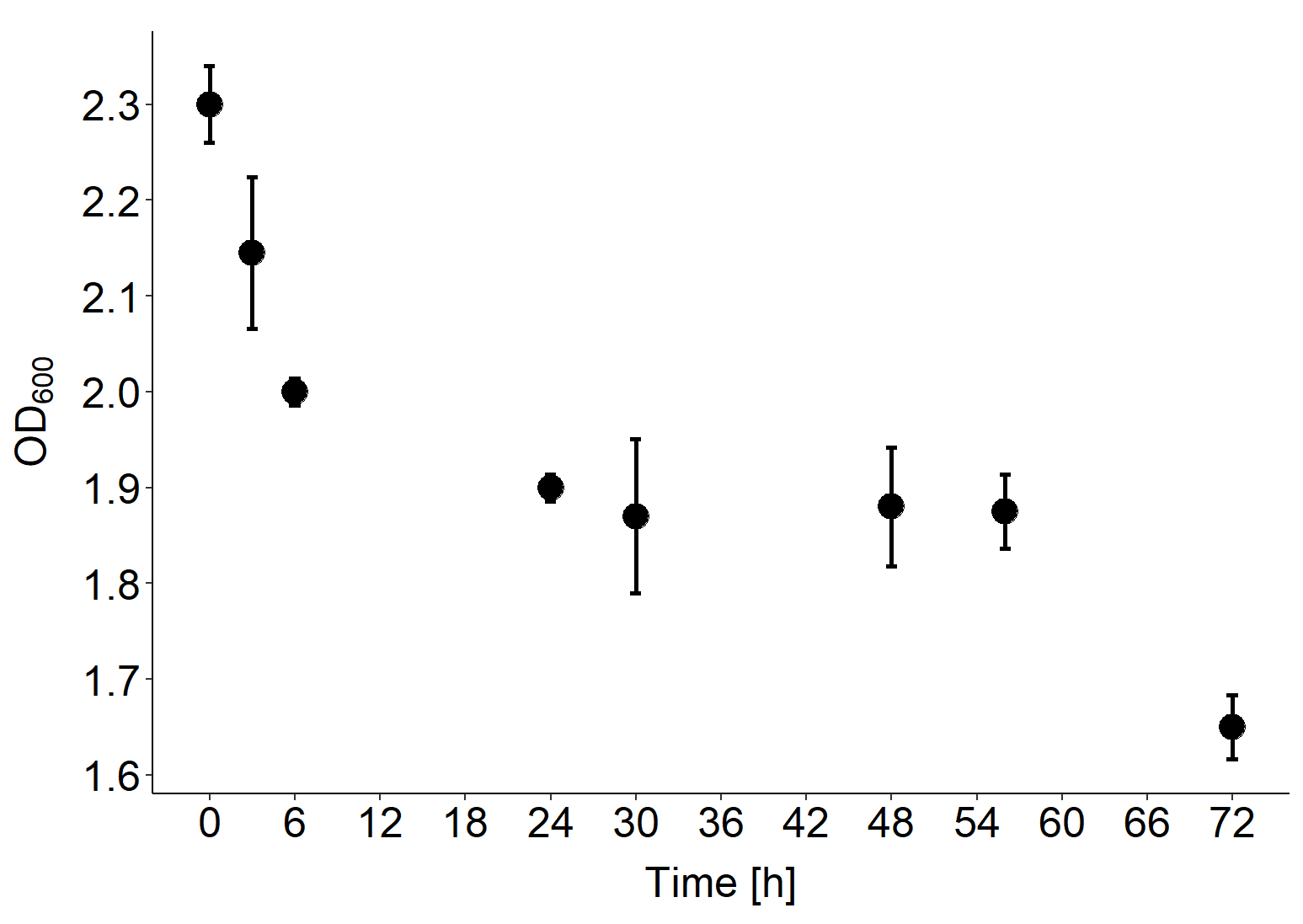


Figure S 9 Change in cell density during a representative biotransformation (second phase) in a solution containing 500 mM pyruvate. The pH was kept constant using a pH-control with 0.5 M HCl.
